# Case report: Complex perianal fistula treated with fistula laser closure (FILAC) and suction catheter

**DOI:** 10.1016/j.ijscr.2021.106085

**Published:** 2021-06-11

**Authors:** Franky Mainza Zulkarnain, Daniel Ardian Soeselo, Suryanto, Gregorio Gavriel Singgih

**Affiliations:** a Department of Surgery, Pondok Indah Puri Indah Hospital, Jakarta, Indonesia; b Department of General Surgery, School of Medicine and Health Sciences, Atma Jaya Catholic University of Indonesia, Jakarta, Indonesia; c School of Medicine and Health Sciences, Atma Jaya Catholic University of Indonesia, Jakarta, Indonesia

**Keywords:** Complex fistula ani, Fistula laser closure (FILAC), Case report

## Abstract

**Introduction and importance:**

A patient presented with complex perianal fistula treated with fistula laser closure (FILAC) combined with suction catheters.

**Case presentation:**

Male, 29 years old, presented in our department, presented with complex perianal fistula with a history of fistulectomy and tight seton for 6 months in another health facility. Intraoperative findings were a tract of 4,5 cm long, 4 external openings, and 1 internal opening. Definitive treatment of Fistula Laser Closure (FILAC) with 13-watt power laser diode produces by BIOLITEC German.

**Conclusion:**

The patient recovers within 6 months. Promising results have been shown by combining FILAC and suction catheter for complex perianal fistula.

## Highlights

• Complex perianal fistula is a hard-to-treat diagnosis, careful approaches for its complication are needed.
• FILAC is a new sphincter-saving technique, Combination with a suction catheter promotes FILAC to achieved primary closure.
• FILAC procedure was chosen because it is a safe and effective treatment for transphinteric anal fistula
• Promising result have been shown by combining FILAC and suction catheter for complex perianal fistula

## 1 Introduction and importance

A complex perianal fistula is a hard-to-treat diagnosis, requiring careful approaches. High risk for complication such as fecal or flatus incontinence and recurrences, causes the needs for advance procedure. FILAC was chosen for its safety and effectiveness to treat transsphinteric anal fistula. FILAC is a new sphincter-saving technique that uses laser diode to obliterate fistula tract. Combination with a suction catheter promotes FILAC to achieved primary closure.

## 2 Case presentation

**Fig. 1:**
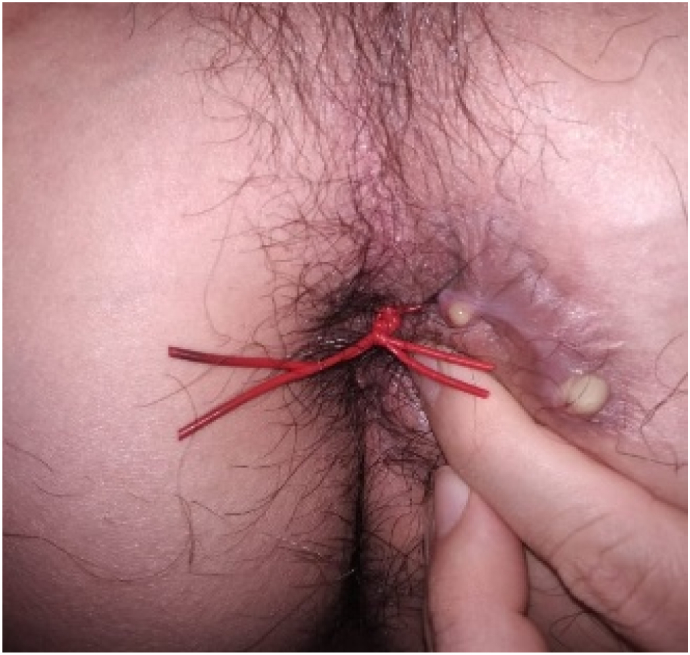
Multiple openings with a seton applied in one of the branches.

**Fig. 2:**
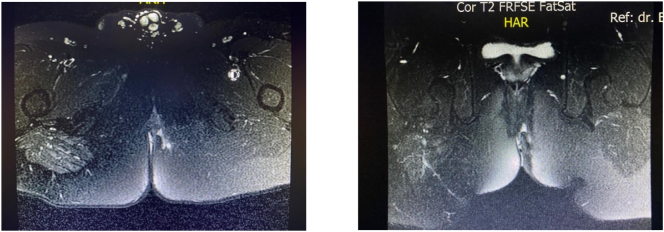
MRI perianal findings.

Male, 25 y.o., presented with a chief complaint of multiple openings around the anus, with a discharge of fluid and feces. The patient has a history of fistulectomy and seton application in another health facility with no satisfactory progress of healing for 6 months, therefore the patient decided to come to our department (Fig. 1). There are no prior history of drug use, familial diseases, and chronic infection. Laboratory findings showed a normal hemoglobin level (14.4 mg/dL), hematocrit (44%), erythrocyte (5.1 million), and a leukocytosis (11.8 thousand). MRI findings confirm a complex fistula with multiple tracts (Park Classification of transsphinteric fistula with secondary tract; St. James University Classification of Grade IV Transsphinteric fistula with ischioanal abscess). The main tract was 4,5 cm long, with internal opening diameter of 6.3 mm and external opening diameter of 5.1 mm and maximum diameter of 9 mm of the tract and seton application in one of the branches accompanied by tubular subcutaneous edema towards left ischiogluteal (Fig. 2).

**Fig. 3:**
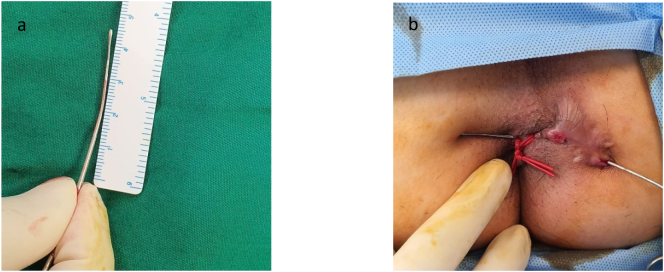
Fistula exploration findings. (a) Main tract of the fistule measuring 4.5 cm long; (b) seton application in one of the branches.

**Fig. 4:**
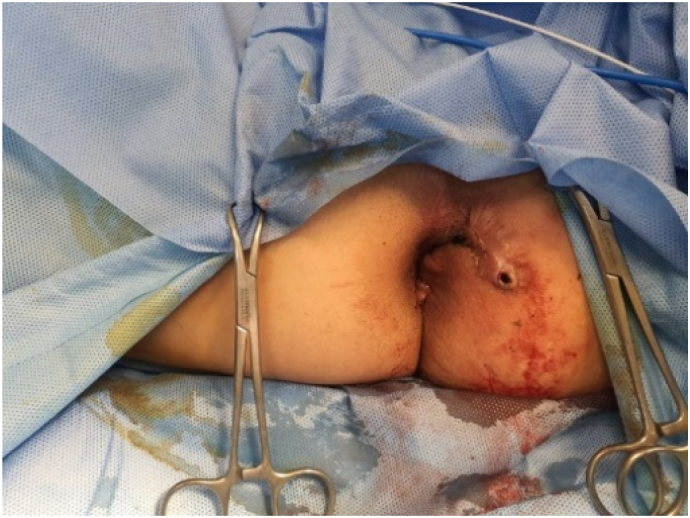
Seton was removed and after FILAC.

**Fig. 5:**
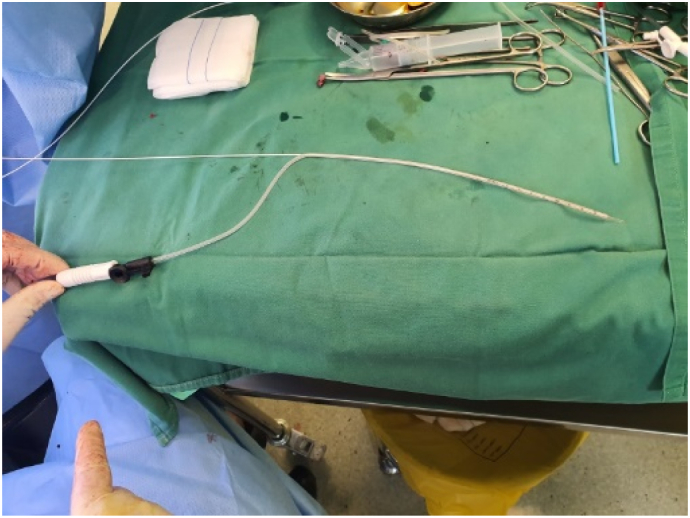
Combination of laser fiber and suction catheter.

The patient undergoes general anesthesia and lithotomy position. Fistula exploration discovers the main tract of 4.5 cm long with four branches and a seton (Fig. 3a–b). Seton was removed (Fig. 4), and tract curettage is done with a cytobrush then cleanse with H_2_O_2_ and NaCl 0.9%. Next, the laser fiber is inserted into a 10 F suction catheter connected with a suction machine of 20 mm Hg pressure (Fig. 5). This combination is intended to create a negative pressure and vacuum effect to minimize the tract's diameter and creating a dry environment for the laser to cut through without any fluid blockage. Before the laser was used, internal opening was closed using 2-0 vicryl. Laser movement began from internal opening until external opening with 13-watt laser power continuous mode, 1 mm/s speed, and cross checking is done every 1 cm to confirm tract closure. This process is applied to other branches. The procedure was done by surgeon Franky Mainza Zulkarnain hence, we called this procedure *Mainza* method. Post-operative intervention includes 2 g of 1st generation Cephalosporin as antibiotics prophylaxis and 50 mg of dexketoprofen intravenously for analgetic.

**Fig. 6:**
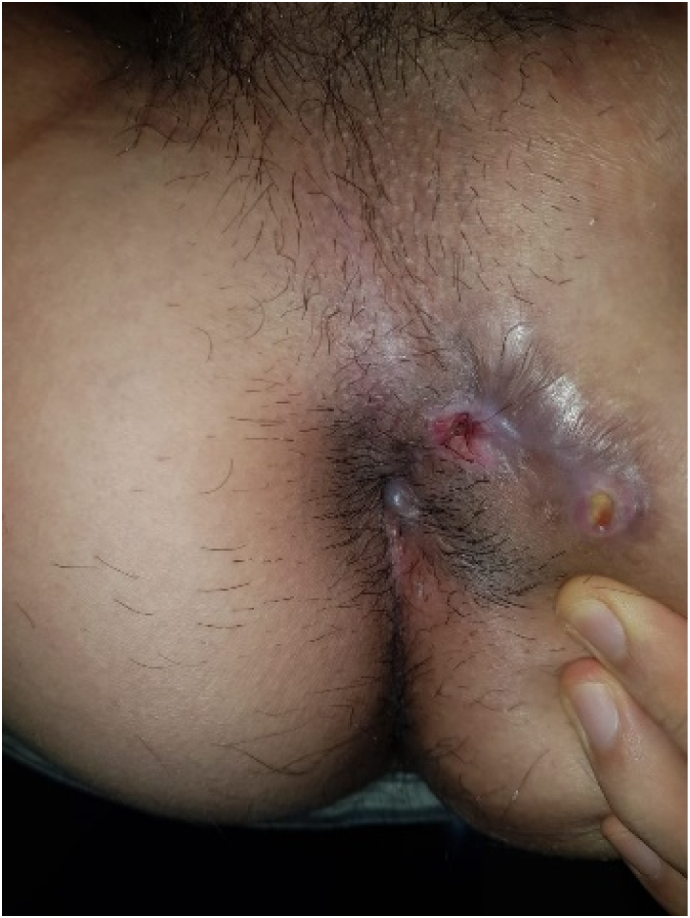
After 3 weeks.

**Fig. 7:**
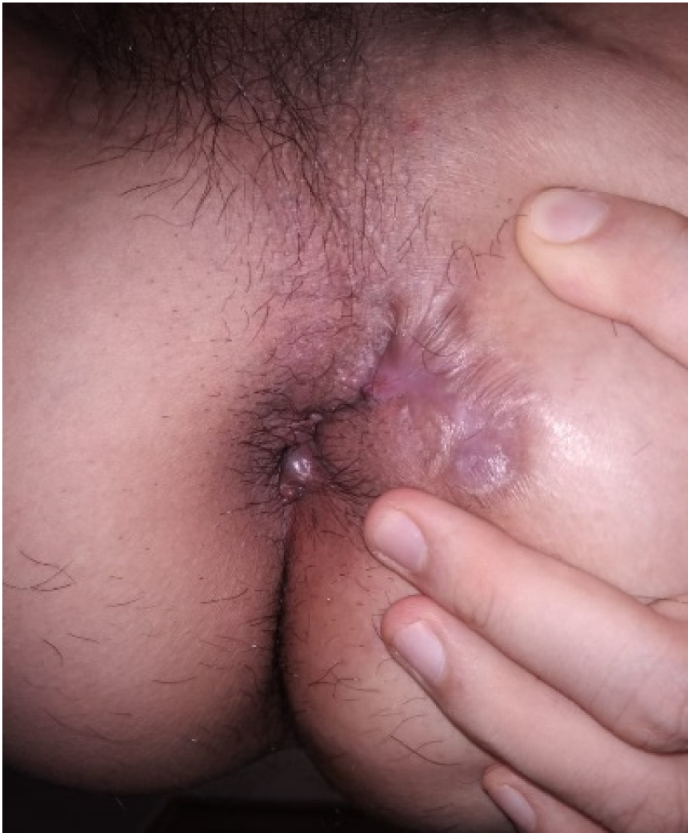
After 8 weeks.

**Fig. 8:**
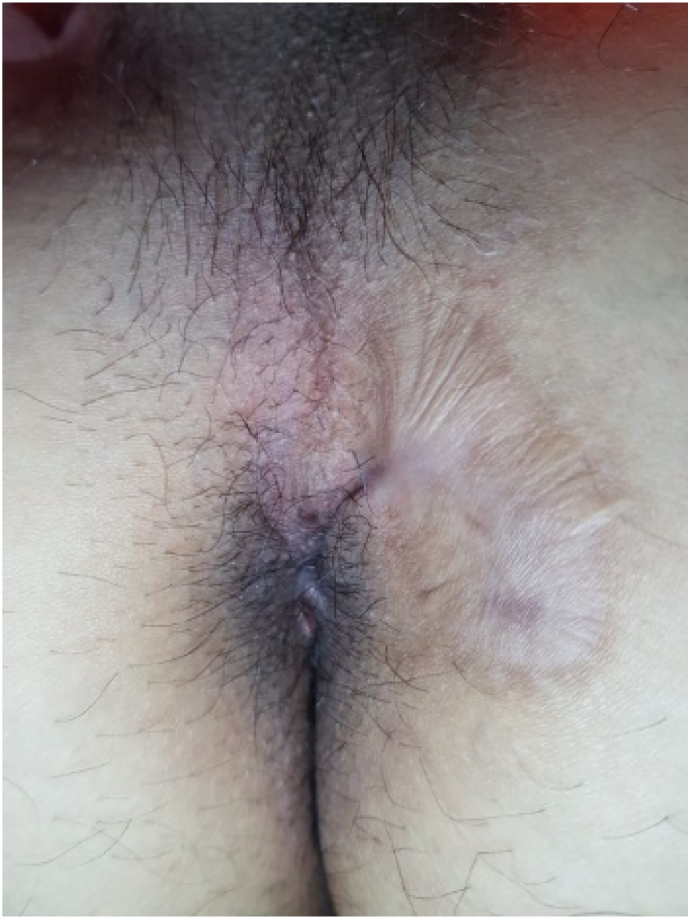
After 6 months.

The patient was hospitalized for one day and discharged by the next day with visual analogue score (VAS) of 2/10. Patient was educated for doing sitz bath at home. A complaint of mild discomfort, exudate fluid discharge with no feces, and localized edema is reported for the first two weeks and decreased at the third week (Fig. 6). Daily activities can be done with no hindrance. Eight weeks post-operation, there is still minimal fluid discharged, no new tract, and no foul-smelling fluid has complained (Fig. 7). From the first month until the fifth month of the treatment, the patient claims an appearance of small bumps that burst on their own and healed. Six months post-operation, wound closure is achieved, and for sixteen months after, patient did not complain of any new openings (Fig. 8).

## 3 Clinical discussion and conclusion

Fistula perianal is a tract that connects perianal skin to the anal canal. The fistula tract is formed by an abscess that results from an obstruction of an anal gland. This obstruction causes chronic infections and epithelialization of the abscess drainage tract [1]. Complex fistula perianal is defined by those with more muscle involvement, or anterior fistulas in female patients, as well as recurrent fistulas, suprasphincteric fistulas, extrasphincteric fistulas, horseshoes fistulas, fistulas associated with irritable bowel disease, transsphincteric fistulas that involve greater than 30% of the external sphincter and those associated with pre-existing fecal incontinence, inflammatory bowel disease, radiation, malignancy, or chronic diarrhea [1,2]. Risk factors for recurrences includes, anatomy of the fistula (Park Classification), lack of comprehensive post-operative assessment, flaws of the surgeon, poor choice of operation, and lack of post-operative care [3].

Nowadays, there are many options for complex fistula perianal treatment, such as ligation of the intersphincteric fistula tract (LIFT), fistula clip closure, draining seton, cutting seton, fistula plug, fistulotomy, and endorectal advancement flap [2]. Complications among this treatment are bleeding, pain, infections, fecal and flatus incontinence. In this case, FILAC procedure was chosen because it is a safe and effective treatment for transphinteric anal fistula [4].

FILAC was developed using a newly invented radial emitting laser probe (“FiLaC™”, Biolitec, Germany) to break the fistula epithelium and to obliterates the remaining fistula track simultaneously by a photothermal effect with coincident closure of both the internal and external fistula orifices [5,6]. Primary closure of the fistula tract is achieved by using a diode laser. Energy emitted by the laser causes shrinkage of tissue and progressive sealing of fistulas [7]. Utilization of the suction catheter created a dry environment for the laser to be effectively cut through the fistula tract. Additionally, vacuum effect forms the suction is achieved and cause negative pressure that promote the tract shrinkage. These two concurrently assisted the laser probe to attain primary closure. Common complications after FILAC was pain and discomfort, minor bleeding, fever, and late abscess [8]. Recovery is achieved within 6 months with no complication reported. Promising result has been shown by combining FILAC and suction catheter for complex perianal fistula. This case report has been reported in line with the SCARE 2020 guideline criteria [9].

## Informed consent

Written informed consent was obtained from the patient for publication of this case report and accompanying images. A copy of the written consent is available for review by the Editor-in-Chief of this journal on request.

## Ethical approval

There is no ethical approval, for this is a case report.

## Funding

This research did not receive any specific grant from funding agencies in the public, commercial, or not-for-profit sectors.

## Guarantor

Franky Mainza Zulkarnain.

## Research registration number

None.

## CRediT authorship contribution statement

Franky Mainza Zulkarnain – writing paper
Daniel Ardian Soeselo – review, writing paper
Suryanto – writing the paper
Gregorio Gavriel Singgih – writing the paper.

## Declaration of competing interest

There is no conflict of interest.
